# Man-Power and Men with Defective Physique

**Published:** 1918-03-02

**Authors:** 


### MAN-POWER AND MEN WITH DEFECTIVE PHYSIQUE.

No medical man who has had any experience doubts that a great number of men are dragged into the Army, where they are useless, from civil occupations in which they were useful, and that many men are retained in the Army not earning their pay or effective for defence or attack who would be of great value in some civilian occupation. It is difficult to avoid this. The greatest possible pressure is exercised on all those responsible for getting men into the Army to pass all it is possible to pass, and the result is a man is passed "He may do." It is not good policy to try to make soldiers of the doubtful. Take a "doubtful" agricultural labourer as an instance. He can plod along ten or twelve hours a day on a farm at his own pace. He will milk six or seven cows morning and evening, he will thatch a rick, spread dung, take a turn at threshing, and keep well and be of service to his country. But take that man and subject him to the strain and stress of a soldier's life, the exposure, the irregular hours, the need for swift straining action, and down he goes. To hospital he goes on full pay, for weeks perhaps; then tries again, and to hospital again. Many such men spend two-thirds of their Army time in hospital, and are no good to their country either then or when they are out of hospital.

There is also a large class in our big towns small of bone, weak of muscle because in all their lives they have never worked laboriously and never played strenuous games. They can tend machines; they are often excellent clerks; they serve amiably in shops; but they are not men, real men fit to fight and defend their community. They are products of our civilisation, only alive because the community cares for them and protects them. They are no use to an Army even if they get as far as the front line. They load up the hospitals. A third class is the dear old fellows, of which every division has a few score. Hardy old chaps often; flat-footed gardeners, rheumaticky labourers, confidential butlers, old village characters. Everybody likes them. Everybody tries to save them as much as possible. When the unit moves they are carried on the transport, relegated to dumps, given cadged lifts on lorries, and so they carry on and show as effectives. In reality they are lumber. But many of them would be worth a pound a week doing part-time jobs in a factory or on a farm; a few might be worth more. They could all, at any rate, keep themselves at no expense to the community, whereas now the Army pays them more than they could earn from any other employer, and they are not worth the money.

The whole of these classes might well be in civil life and the Army be the better for the weeding; but who is to do it? The only people who could do it well are able, experienced, wideawake medical men, with knowledge and experience acquired with fighting divisions, and such are required for other purposes. Committees composed of men without plenty of war experience and medical boards of men without war experience and old dug-outs will not be competent to judge. Whoever does the job will have to convince all concerned that because a man has no disease and because a man can do some sort of work in some sort of way it does not follow that that man is fit for the Army or worth keeping in it, and to make it understood that though a paper soldier may stop a bullet he will not do as much to gain victory for us in the Army as he might in civil life, and is not worth his pay and pension as a fighting unit.

